# STEAM-SASHA: a novel approach for blood- and fat-suppressed native T1 measurement in the right ventricular myocardium

**DOI:** 10.1007/s10334-023-01141-8

**Published:** 2024-01-12

**Authors:** Malte Roehl, Miriam Conway, Sarah Ghonim, Pedro F. Ferreira, Sonia Nielles-Vallespin, Sonya V. Babu-Narayan, Dudley J. Pennell, Peter D. Gatehouse, Andrew D. Scott

**Affiliations:** 1 Cardiovascular Magnetic Resonance Unit, Royal Brompton Hospital, Guy’s and St Thomas’ NHS Foundation Trust, Sydney Street, London, UK; 2 National Heart and Lung Institute, Imperial College London, London, UK

**Keywords:** Cardiovascular magnetic resonance, Tissue characterization, T1 mapping, Right ventricle, Artefacts

## Abstract

**Objective:**

The excellent blood and fat suppression of stimulated echo acquisition mode (STEAM) can be combined with saturation recovery single-shot acquisition (SASHA) in a novel STEAM-SASHA sequence for right ventricular (RV) native T1 mapping.

**Materials and methods:**

STEAM-SASHA splits magnetization preparation over two cardiac cycles, nulling blood signal and allowing fat signal to decay. Breath-hold T1 mapping was performed in a T1 phantom and twice in 10 volunteers using STEAM-SASHA and a modified Look-Locker sequence at peak systole at 3T. T1 was measured in 3 RV regions, the septum and left ventricle (LV).

**Results:**

In phantoms, MOLLI under-estimated while STEAM-SASHA over-estimated T1, on average by 3.0% and 7.0% respectively, although at typical 3T myocardial T1 (T1 > 1200 ms) STEAM-SASHA was more accurate. In volunteers, T1 was higher using STEAM-SASHA than MOLLI in the LV and septum (*p *= 0.03, *p *= 0.006, respectively), but lower in RV regions (*p *> 0.05). Inter-study, inter-observer and intra-observer coefficients of variation in all regions were < 15%. Blood suppression was excellent with STEAM-SASHA and noise floor effects were minimal.

**Discussion:**

STEAM-SASHA provides accurate and reproducible T1 in the RV with excellent blood and fat suppression. STEAM-SASHA has potential to provide new insights into pathological changes in the RV in future studies.

**Supplementary Information:**

The online version contains supplementary material available at 10.1007/s10334-023-01141-8.

## Introduction

Myocardial T1 mapping in the left ventricle is now a well-established methodology, used routinely in clinical MRI centres worldwide [1, 2]. However, despite potential clinical applications of T1 mapping in the right ventricular (RV) myocardium, such as the assessment of diffuse fibrosis in repaired tetralogy of Fallot [3–5], T1 studies targeting the RV are few and far between [6, 7]. The macroscopic anatomy of the RV is complex, with a thin compact wall and a thicker network of trabeculations with blood filling the spaces within the network. In addition, a subject-specific proportion of the RV epicardium is covered in a layer of fat with variable thickness [8]. The bright signal from both fat and blood in typical T1-mapping sequences, with the limited spatial resolution available means that obtaining T1 measurements in the RV myocardium without contamination from blood and fat signal is difficult. To reduce these partial volume effects and provide accurate measures of RV myocardial T1, substantial improvements in either spatial resolution or suppression of unwanted signals are required. Several studies have attempted to improve spatial resolution [6, 7], but here we introduce a novel technique which targets the contaminating blood and fat signals.

Stimulated echo acquisition mode (STEAM) sequences are commonly used to provide diffusion tensor cardiovascular magnetic resonance (DT-CMR) in the in-vivo myocardium without the effects of bulk cardiac motion [9, 10]. Such techniques split the stimulated echo signal preparation over two cardiac cycles, with the two parts of the preparation each happening at the same trigger time in the two cardiac cycles. During the one cardiac cycle mixing time between the two parts of the stimulated echo signal preparation, the partially prepared magnetisation is stored along the longitudinal axis and T1 recovery causes the stored magnetisation to decay with T1. Due to the short T1 of fat compared to myocardium or blood, fat signal is well suppressed in STEAM images. Furthermore, STEAM requires spoiler gradients in both stages of the signal preparation to avoid unwanted echoes and free induction decay contaminating the desired stimulated echo. These spoiler gradients introduce substantial sensitivity of the sequence to motion between the application of the two gradients, which suppresses the signal of the moving blood in the heart and blood vessels. The stimulated echo is then sampled, most commonly using a single shot EPI method to maximise imaging efficiency.

Here we aim to leverage the intrinsic blood nulling and excellent fat suppression of single-shot STEAM EPI acquisitions for accurate and reproducible T1 mapping in the RV myocardium. We introduce the STEAM-SASHA (saturation recovery single shot acquisition) technique, which combines a saturation pulse T1 preparation with single shot STEAM EPI to provide blood and fat suppressed T1 measurements. We use phantom measurements to investigate accuracy then healthy volunteer studies to assess blood suppression and reproducibility, in both cases comparing to a reference modified Look-Locker imaging (MOLLI) technique.

## Materials and methods

### STEAM-SASHA

**Fig. 1:**
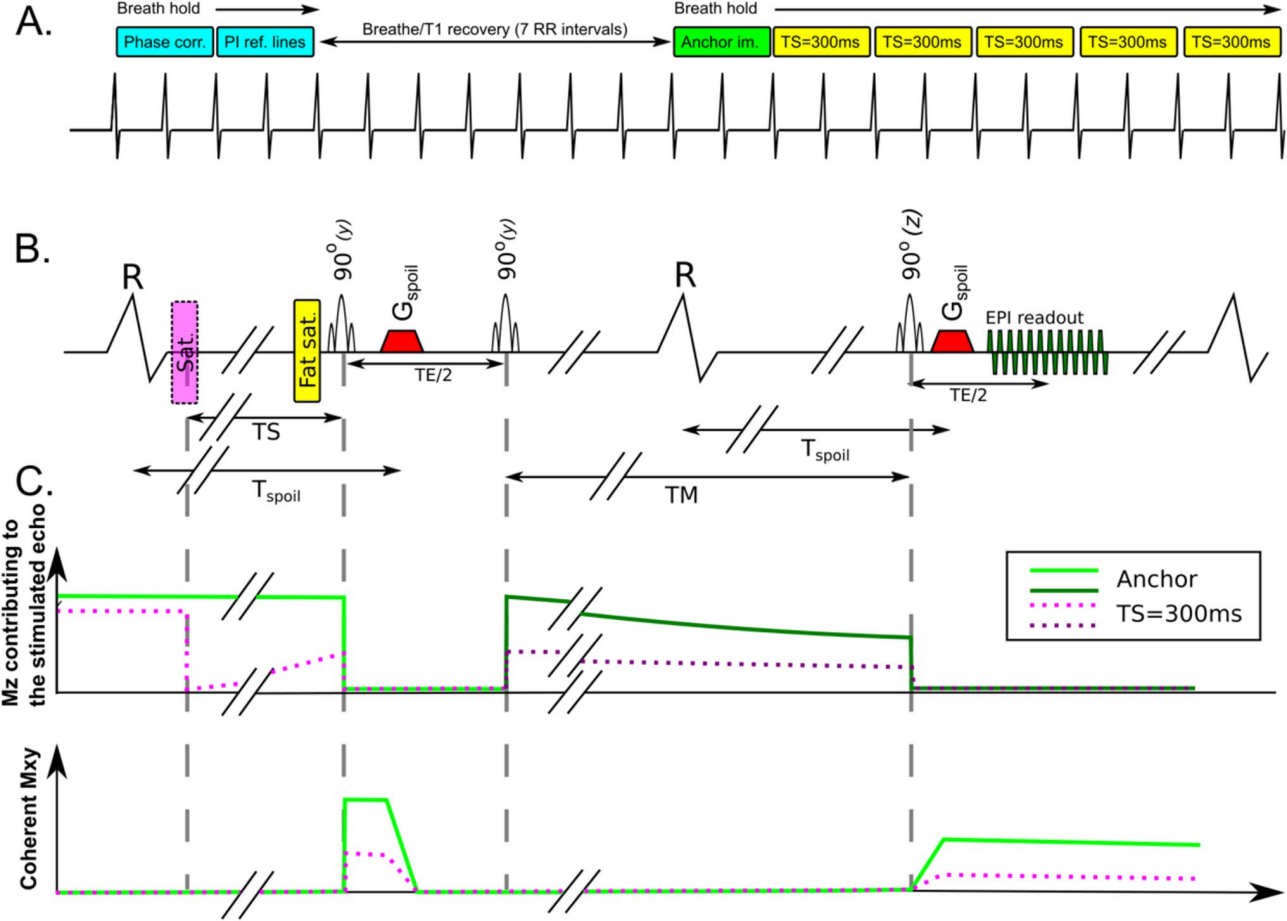
Each repeat of the sequence provides 1 anchor image (without the saturation pulse) and five TS=300 ms images **a** acquired across 2 breath holds. The first of these 2 breath holds is used to acquire the preparation scans and is followed by a T1 recovery period before the anchor image is acquired. The STEAM EPI sequence requires 2 heartbeats per image **b**. The dimension of the region contributing signal to the image in the phase encode direction is reduced by making the first two RF pulses slice selective in the phase encode direction (as denoted by “(*y*)” above the pulses), therefore allowing a shorter EPI readout. The third RF pulse is slice selective in the through plane direction (“(*z*)”). Non-stimulated echoes (i.e. gradient echoes and stimulated anti-echoes) are removed using spoiler gradients (*G*_spoil_) that run at the same delay (*T*_spoil_) from the ECG R-wave in both heartbeats. This spoiler timing avoids bulk cardiac motion related signal loss due to movement between the gradients, but spoils signal from the moving blood. T1 weighting is provided by a saturation pulse with TS=300 ms. A schematic plot of the evolution of the magnitude of the longitudinal (Mz) available to generate the stimulated echo and coherent transverse (*M*_*xy*_) magnetisation in water is shown for one repetition of either the anchor or TS=300 ms STEAM EPI **c** and a corresponding plot showing *M*_*z*_ for fat is provided in Fig. S1. Longitudinal magnetisation after the second RF pulse is shown in a darker colour to denote that we do not show the recovering component that does not contribute to the stimulated echo, but would generate a free induction decay signal if it were not for the second *G*_spoil_ gradient

Similar to STEAM sequences used for DT-CMR [9, 10], STEAM-SASHA splits signal preparation over two consecutive cardiac cycles with a single-shot EPI readout in the second cardiac cycle (Fig. 1a). The first two of the 90° RF pulses play out after the first R wave and the final 90° pulse runs after the next R wave. To remove the stimulated anti-echo and gradient echo from the image, spoiler gradients are played out after the first and third RF pulses (see [11, 12] for more details on generation of stimulated echoes). The effective diffusion encoding of the spoiler gradients is small (*b* = 100 smm^−2^) and the orientation of the spoiler gradients was rotated between sequence repeats to minimise any directional bias effects. Cardiac motion related signal loss was avoided by running the two spoiler gradients at identical timings in the two consecutive cardiac cycles (*T*_spoil_).

A shorter EPI readout [13] was enabled via a reduced phase field of view technique. The RF pulses that run in the first cardiac cycle are selective in the phase-encoding direction, while the third is selective through plane, making the field of view contributing to the stimulated echo smaller in the phase encode direction. Fat signal is suppressed via using a fat saturation pulse which reduces the longitudinal fat magnetisation available to be tipped into the transverse plane by the first RF pulse and contribute the stimulated echo. Further fat suppression is provided by the rapid longitudinal recovery towards equilibrium (and therefore rapid decay of the fat signal available to contribute to a stimulated echo) during the mixing time (TM) of any residual longitudinal fat magnetisation not supressed by the saturation pulse (see Fig. S1). When saturation pulses were applied, they were run immediately after the R-wave, and used a fixed saturation time of TS=300 ms before the first of the 90° RF pulses used for STEAM. This saturation time was chosen to place the EPI readout near peak systole when the myocardial wall is thickest.

Each sequence repeat was acquired over two breath holds (Fig. 1b). During the first breath hold two stimulated echoes (4 RR-intervals) were acquired for EPI phase correction data and parallel imaging reference data. This was followed by 7 RR-intervals for T1 recovery time (while the subject breathed in then out again). During the second breath hold (12 RR-intervals) STEAM-SASHA acquires an anchor image with no saturation pulse then five TS=300 ms images. The anchor image provides the reference signal intensity as the longitudinal magnetisation is allowed to fully recover before the stimulated echo RF pulses begin (Fig. 1c). The other sequence parameters for STEAM-SASHA included TE=36 ms, TR=2RR-intervals, 2.8 × 2.8 mm^2^ acquired resolution, 8 mm slice thickness, receiver bandwidth = 312.5 kHz, 360 × 270 mm^2^ imaged field of view, 47 ms EPI train duration, 6/8 partial Fourier and factor 2 SENSE.

A vendor supplied saturation pulse module was used consisting of three non-selective RF pulses, each with a flip angle of nominally 90° (see section “Discussion”) and a total duration of 11.5 ms.

Images were also acquired with identical sequence parameters using the same coil sensitivity reference scans but without the imaging RF pulses, providing noise images for measuring background signal levels. Magnitude STEAM-SASHA images were reconstructed at the scanner using a vendor supplied reconstruction.

### MOLLI sequence

For comparison with the STEAM-SASHA data, 5b(3b)3b MOLLI data was acquired during a breath hold using a vendor supplied product sequence. The sequence used a minimum TI = 100 ms (increment 80 ms), balanced steady-state free precession readout, 2.4 × 2.0 mm^2^ acquired resolution (phase × frequency encode), 307 × 360 mm^2^ field of view, 8 mm slice thickness, 208.3 kHz receiver bandwidth, 7/8ths partial Fourier, factor 2 GRAPPA, flip angle = 20°, TE = 1.0 ms and TR = 2.4 ms. Images were acquired at peak systole for each subject based on short axis cine data. T1 maps were generated at the scanner using the online (“Myomaps”) vendor supplied software.

### Phantom validation

All phantom and in vivo imaging was performed on a 3T Siemens Vida using anterior and spine coil arrays. Validation of T1 measures was performed in the T1MES phantom [14]. Data were acquired using both STEAM-SASHA and MOLLI techniques. The ‘slow scanning’ data from [14] were acquired by our group in the same room with the same temperature settings and are therefore used as a gold standard.

### In vivo study

The healthy volunteer cohort consisted of 10 subjects (4 female, mean age 35 years, range 24–56 years) recruited with written informed consent according to local ethical approvals. A single mid-ventricular short axis RV slice was identified using balanced steady state free-precession cine acquisitions (typical acquired in-plane resolution 1.4 × 1.7 mm^2^). This slice was oriented to place the right ventricular free wall as close as possible to perpendicular to the imaged slice in multiple long axis views of the RV and used for the subsequent native T1 measurements. STEAM-SASHA data were acquired using TS=300 ms via a saturation pulse applied immediately after the R-wave trigger. For the MOLLI data, the trigger delay was set so that the central *k*-space data were acquired at peak systole. In both cases, systolic triggering was chosen as the myocardium is thickest in this cardiac phase and much of the blood within the trabeculations is expelled, reducing partial volume effects in the mid-myocardium. The adjust volume was placed over the left and right ventricle in the imaged short axis plane with minimal extension beyond. For each scan the STEAM-SASHA acquisition was repeated 8 times (giving 8 anchor images and 40 TS=300 ms images) and the MOLLI acquisition was repeated twice, as is performed as standard in research protocols at our institution to reduce T1 variability. Therefore, each T1 mapping acquisition required 8 short (4 cardiac cycles) and 8 long (12 cardiac cycles) breath holds for STEAM-SASHA and 2 breath holds (11 cardiac cycles) for MOLLI.

Healthy volunteer scans were repeated with repositioning in-between (i.e. 4 repeats of the MOLLI sequence and 16 repeats of the STEAM-SASHA sequence total) to provide data for assessing inter-study reproducibility.

### Analysis

**Fig. 2:**
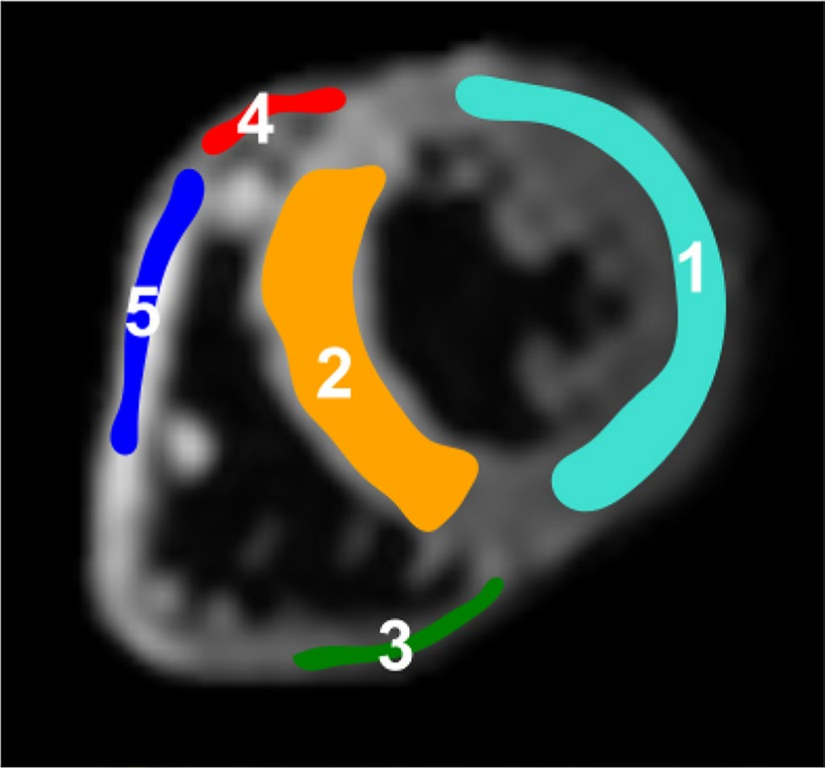
An example of the approximate locations of the 5 regions of interest used in the analysis. (1) left ventricle (LV), (2) septum, (3) inferior RV, (4) anterosuperior RV, (5) anterolateral RV overlaid on a STEAM-SASHA anchor image

Custom Python software was developed to process the STEAM-SASHA data. Motion-corrupted images were discarded manually, and images were cropped to a region around the heart. Motion within a breath hold was assumed to be negligible and the images within each breath hold were masked manually to show only the myocardium. The anchor images were rigidly registered (Elastix toolbox) to correct for inter-breath hold motion and the displacements were applied to all images from the same breath hold. Then five regions were defined on the images (see Fig. 2) with inter-breath-hold (i.e. for each breath hold separate) adjustment of the regions for changes in myocardial shape. Pixelwise and regionwise T1 values were calculated according to: $T1\left[{\text{ms}}\right]=\frac{-300{\text{ms}}}{{\text{ln}}\left(1 - {S}_{TS 300{\text{ms}}}/{S}_{{\text{Anchor}}}\right)}$, where *S*_*TS*300ms_ and *S*_Anchor_ are the mean saturation recovery and anchor image signal intensities respectively. Regions were defined avoiding myocardial border pixels.

Matching regions were defined on the equivalent MOLLI T1 maps in ImageJ (National Institutes of Health). MOLLI T1 values were averaged over each region of interest and over both sequence repetitions in each scan.

As the number of subjects was limited to 10, non-parametric statistics were used as normality could not be reliably assessed. Statistical comparisons were performed using a two-sided Wilcoxon sign-rank test with *p *< 0.05 considered significant.

The processing was repeated by one observer and then by a second observer to provide data for inter- and intra-observer variability. Data from the initial and repeat acquisitions were used to assess inter-study reproducibility. Reproducibility was assessed using Bland–Altman analysis and coefficient of variation (defined as the standard deviation of the difference between scans divided by the mean of the measurement).

To evaluate the number of STEAM-SASHA averages required for stable T1 values, the in vivo data were reanalysed using a variable number of averages (i.e. using *N* anchor images, and 5× N TS=300 ms images, where *N *= 2 − 7).

Background noise can result in a bias in parametric maps when the signal in the weighted images approaches the background noise level [15]. As the STEAM method is inherently a low SNR method, we evaluated the signal level in a septal region of interest from all 20 healthy volunteer scans in the noise-only images, the TS = 300 ms and the anchor images. Signal values were quantified as the mean within the region of interest for the TS = 300 ms and anchor images, and by calculating the signal intensity at the maximum of a Rayleigh distribution fitted to a histogram of the pixel signal intensities in the same region of interest in the noise only images. Blood suppression in the STEAM-SASHA data was evaluated using the signal from a region of interest in the RV blood pool in the noise only images, TS = 300 ms images and anchor images. In all the blood signal suppression measurements, we quantified the signal using the Rayleigh distribution fitting method.

## Results

### Phantom validation

**Fig. 3:**
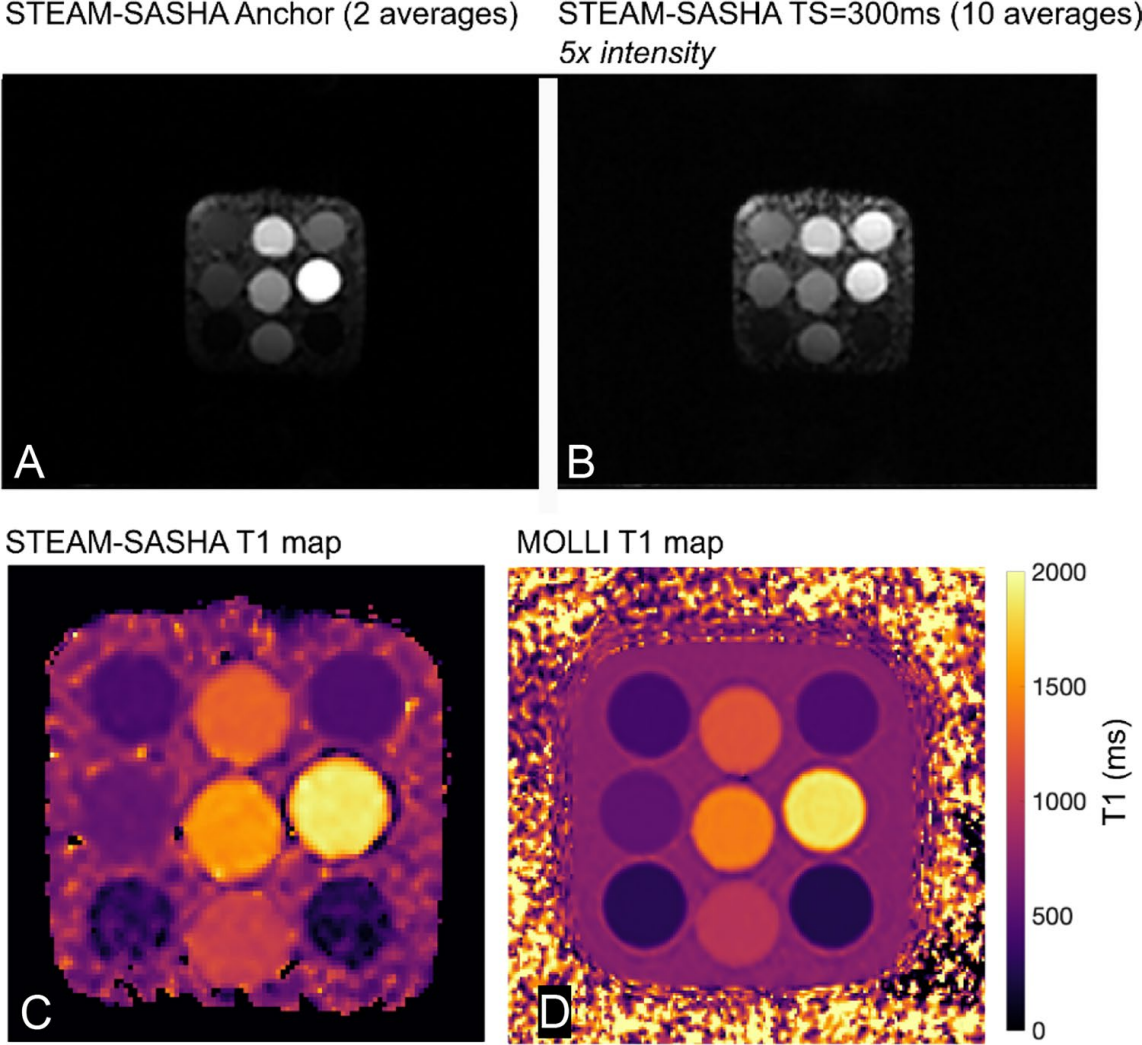
Example averaged anchor (**a**) and TS = 300 ms (**b**) magnitude images from STEAM-SASHA. T1 maps are shown for STEAM-SASHA (**c**) and MOLLI (**d**). STEAM-SASHA data were acquired with 2 sequence repeats/4 breath holds MOLLI data with 2 sequence repeats/2 breath holds

**Fig. 4:**
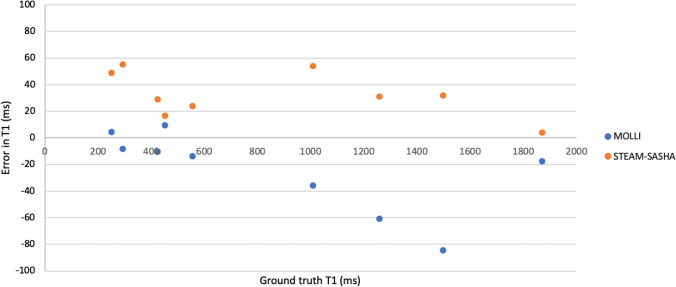
A plot of the error in T1 obtained with MOLLI and STEAM-SASHA in the T1MES phantom. Reference values from [14] acquired using a slow inversion recovery sequence were taken as ground truth. A positive value represents an over-estimation of T1 using the method shown

STEAM-SASHA images from the T1MES phantom, both for the anchor image and the TS = 300 ms image (two sequence repeats: 2 averages of the anchor image and 10 averages of the TS=300 ms images) are shown in Fig. 3, along with the T1 maps from STEAM-SASHA and MOLLI. As expected, due to the inherent SNR inefficiencies of STEAM, the T1 map from STEAM-SASHA appears more noisy than for MOLLI. STEAM-SASHA overestimated T1, while MOLLI underestimated T1 in most tubes, with a mean absolute error 7.0% and 3.0%, respectively (compared to the reference slow inversion recovery values [14], Fig. 4). In the range of T1 values typically expected for the myocardium at 3T in vivo (T1 > 1200 ms), results obtained with STEAM-SASHA had smaller errors than the MOLLI results (absolute STEAM-SASHA errors < 2.5%).

### T1 in healthy volunteers using STEAM-SASHA compared to MOLLI

**Fig. 5:**
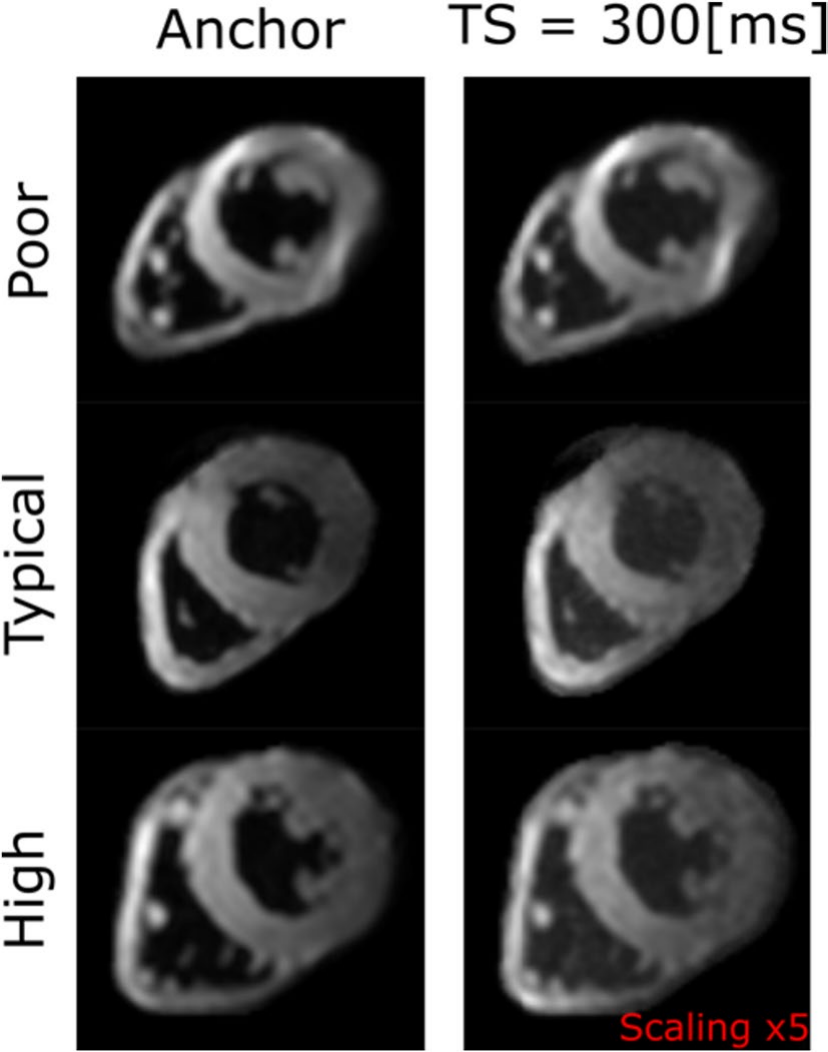
Example STEAM-SASHA images acquired in 3 example subjects with poor, typical and high image quality. Both anchor data (8 averages) and the TS = 300 ms data (40 averages) are shown. The TS = 300 ms data are scaled × 5 in brightness relative to the anchor image data and signal outside the heart has been nulled. Figures S2–S4 show STEAM-SASHA images without averaging

**Fig. 6:**
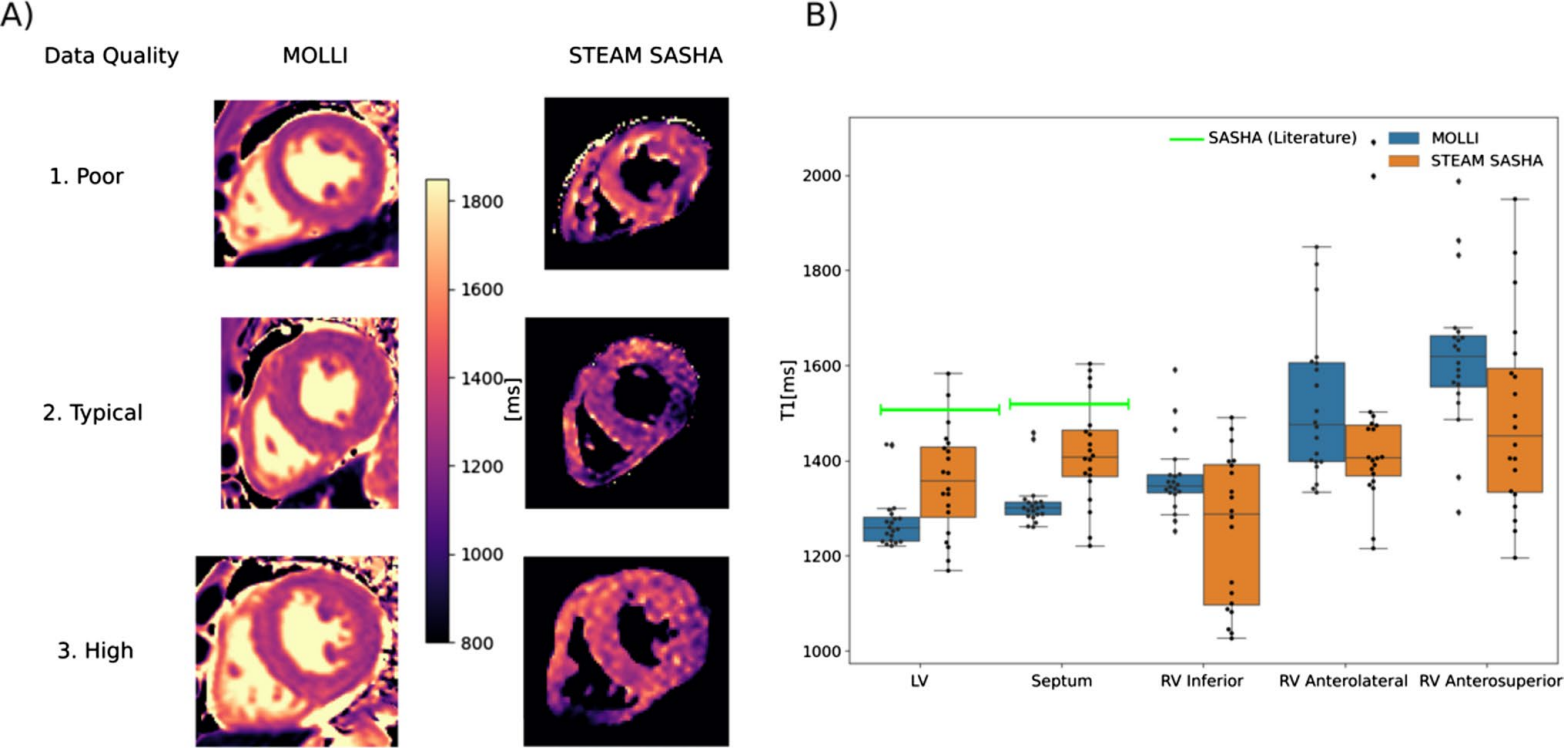
Example in vivo T1 maps calculated from both MOLLI and STEAM-SASHA data (**a**). To show the range in quality of STEAM-SASHA T1 maps, poor, typical and high-quality examples (based on the STEAM-SASHA maps) are shown. As blood is suppressed in STEAM-SASHA, pixels outside the myocardium are nulled in the STEAM-SASHA T1 map. Native T1 obtained with MOLLI and STEAM-SASHA for both scans of each subject are plotted for each of the five regions (**b**). Box plots in **b** show the range and quartiles and reference 3T SASHA values from the literature are shown as green bars [16]. Note the difference in colour scale between the T1 maps shown here and those shown in Fig. 3 for the phantom. Figure S6 shows equivalent Bland–Altman plots for the data in B

All 10 healthy volunteers were successfully scanned twice using both MOLLI and STEAM-SASHA. Mean ± standard deviation of the systolic RV myocardial thickness (measured on the RV short axis bSSFP cine images) was 4.5 ± 1.1 mm in the anterolateral region and 4.9 ± 1.3 mm in the inferior region. Example anchor and TS = 300 ms STEAM-SASHA images from three subjects after averaging are shown in Fig. 5 (the equivalent unaveraged images are shown in Figs. S2–S4). Figure 6 shows example T1 maps obtained using both sequences in three subjects. Figure S5 shows a STEAM-SASHA anchor image with the corresponding bSSFP cine image at peak systole, demonstrating the efficacy of the fat suppression in the STEAM-SASHA sequence in the subject with the most epicardial fat.

Median STEAM-SASHA T1 is non-significantly lower than MOLLI in the RV regions (*p *> 0.05), but higher in the septum and LV (*p *= 0.006 and *p *= 0.03 respectively) (see Fig. 6 and Bland–Altman plots in Fig. S6). For comparison we also plot SASHA values from Weingärtner et al. [16] in Fig. 6 for reference. These reference values are both (septum = 1523 ± 56 ms, LV = 1500 ± 66 ms) higher than the mean values in the corresponding regions in our STEAM-SASHA data (septum = 1422 ± 102 ms, LV = 1360 ± 116 ms regions as defined on Fig. 2). However, the values from Weingärtner et al. [16] are within the corresponding ranges of the STEAM-SASHA values. The variation in T1 between subjects was also greater using STEAM-SASHA than MOLLI in all regions apart from the anterolateral RV. The Bland–Altmann plots shown in Fig. S6, show a significant correlation in the difference in T1 (STEAM-SASHA–MOLLI) vs. mean T1 in the septum and LV ROIs (*R* = 0.69, *p *= 0.03 and *R* = 0.70, *p *= 0.02, respectively), which is consistent with the increasing underestimation in MOLLI T1 with increasing T1 values shown in the T1MES phantom experiments.

### Reproducibility of T1 measures

**Fig. 7:**
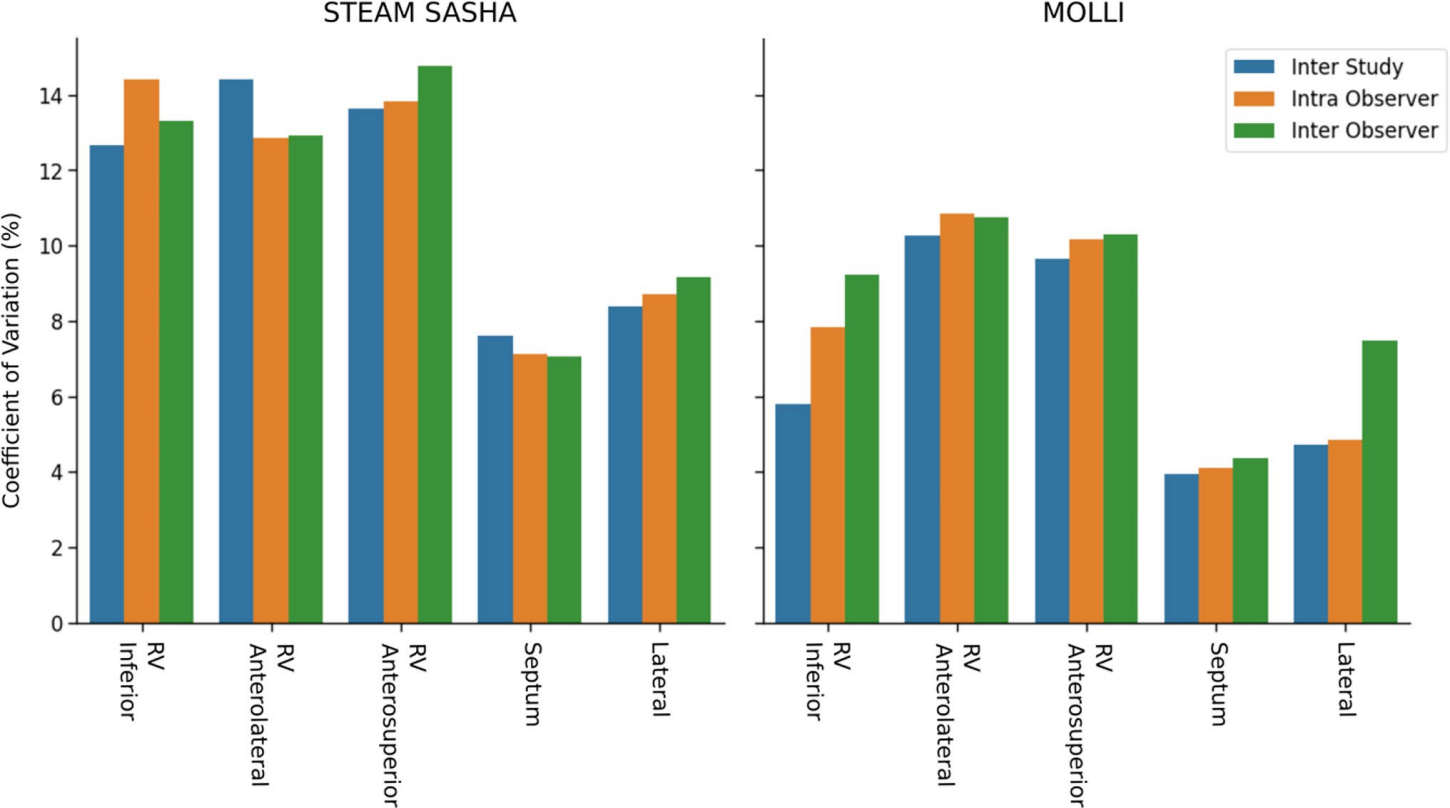
The reproducibility (inter-study, intra-observer, inter-observer, measured as coefficient of variation) for each of the five myocardial regions assessed using both MOLLI and STEAM-SASHA

Intra observer, inter observer and inter study reproducibility were compared using the coefficient of variation (COV) and Bland–Altman analysis. While COV is generally lower using MOLLI and is lower in the LV than the RV regions, COV is less than 15% in all regions (see Fig. 7).

Bias in the results between observers, within observers and between scans is small with the maximum (58 ms) in the anterosuperior RV for inter-study STEAM-SASHA data (Figs. S7–S8). For all reproducibility types, the bias is lower for STEAM-SASHA in the RV anterolateral region than it is for MOLLI.

In the majority of regions for all reproducibility types the 95% confidence intervals in the BlandAltman analysis are larger for the STEAM-SASHA technique, although one notable exception is in the inter-study reproducibility again in the RV anterolateral region where the STEAM-SASHA T1 has smaller limits of agreement than MOLLI.

### Number of averages required for STEAM-SASHA

When comparing to the T1 value obtained with the full 8-average dataset (8 anchor images and 40 TS = 300 ms images) there is generally a reduction in both the variability between scans and the mean error as the number of averages increases (see Fig. S13).

### Noise floor test

**Fig. 8:**
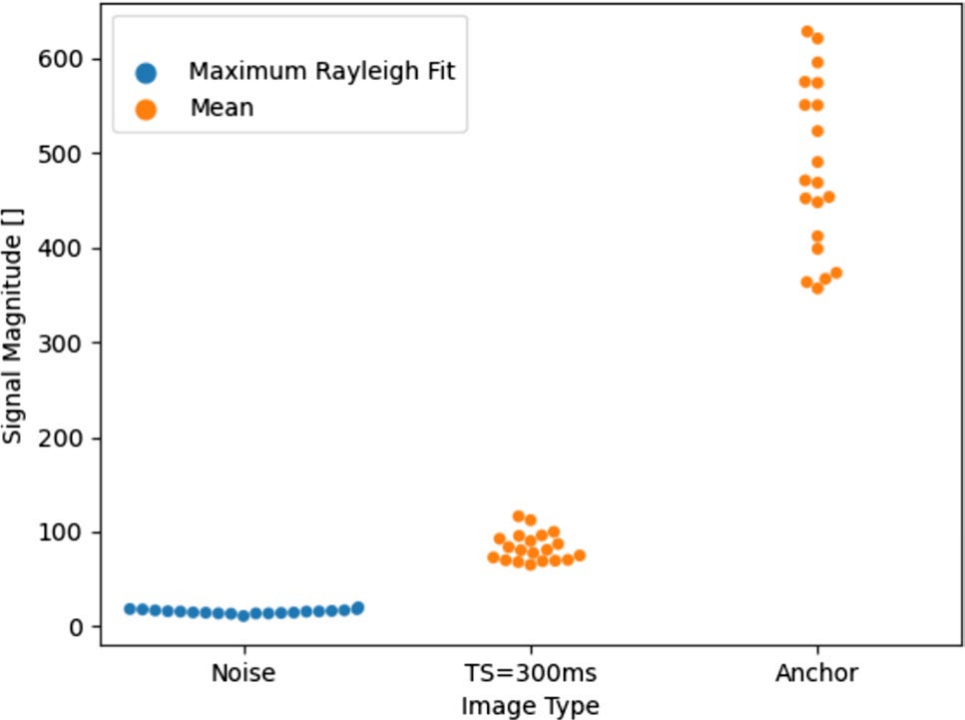
The signal level in a septal region of interest for the noise-only, TS = 300 ms and anchor images in all 20 healthy volunteer scans. While for the TS = 300 ms and anchor images, the mean signal within the region of interest was used, the intensity at the maximum of a Rayleigh distribution fitted to a histogram of the signal intensities within the region of interest was used for the noise measurement

In the TS = 300 ms images, the mean septal image intensity is 96. In the anchor image, the signal in the same region ranges from 380 to 640 (see Fig. 8). In the noise only images, the signal in the TS = 300 ms images is more than four times greater than the noise level and the equivalent signal in the anchor image is more than 19 times greater.

### Blood suppression test

**Fig. 9:**
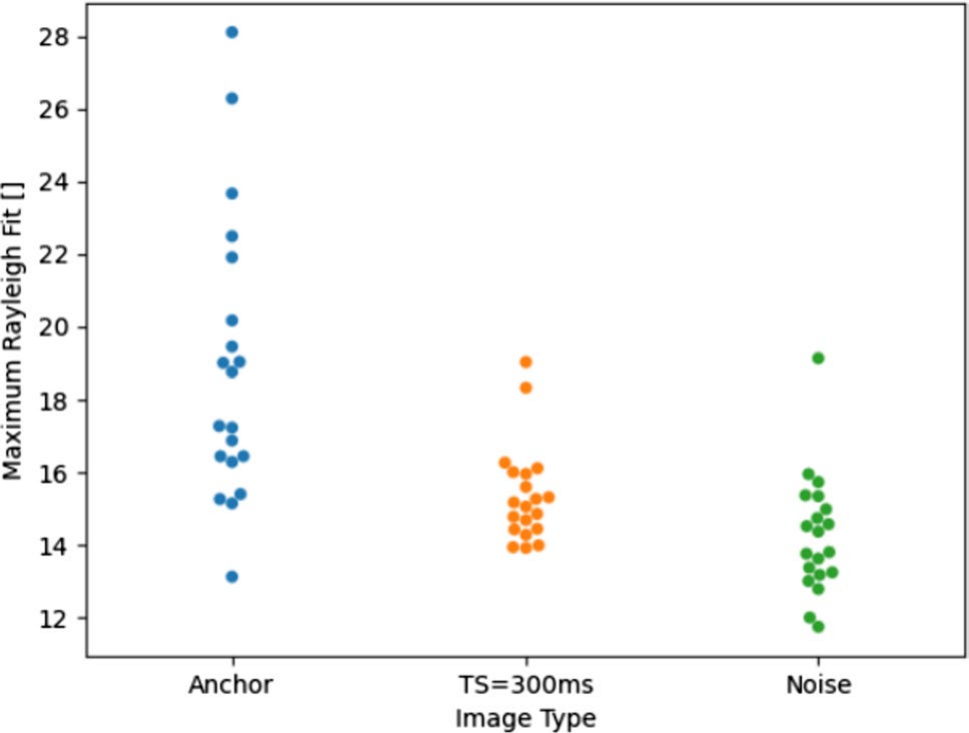
The signal level in the RV blood pool from all 20 healthy volunteer scans from the anchor, TS = 300 ms, and noise-only images. The signal level was calculated by fitting a Rayleigh distribution to the histogram of the signal intensities within the region of interest values and then using the signal level at the maximum of this fitted distribution

Blood is effectively supressed by the STEAM-SASHA method (Fig. 9). The signal in a region of interest in the RV blood pool in the TS = 300 ms images is similar to the signal in the “noise only” data. In the anchor image, the blood pool signal was measured at up to 28 compared to maximum values of approximately 19 in both the noise only and TS = 300 ms data. These values are all low relative to the myocardial signals (see “Anchor” and “TS = 300 ms” data in  Fig. 8).

## Discussion

The STEAM-SASHA sequence suppresses blood and fat signal thereby avoiding partial volume effects and providing accurate and reproducible T1 values. To our knowledge, no SASHA-based RV myocardial T1 values have been published from either healthy subjects or patient cohorts at 3T. Measurements in the T1MES phantom validated the accuracy of STEAM-SASHA derived T1 values and demonstrated that at the values of T1 expected in native myocardium at 3T, STEAM-SASHA values are more accurate than a standard MOLLI sequence. The in vivo results obtained in the LV and septum showing a positive correlation in the STEAM-SASHA–MOLLI bias with increasing T1 are also consistent with this underestimation of T1 at higher T1 values when measured with MOLLI (Fig. S6). Similar trends are observed in the RV inferior and anterosuperior regions, although the correlations fail to reach significance (*p *> 0.05). While T1 values from STEAM-SASHA were consistently longer than values obtained from MOLLI both in the phantom and the LV data in vivo, MOLLI is well known to substantially under-estimate true T1 [14, 17], while providing more precise values. T1 values obtained using STEAM-SASHA were consistent with literature results obtained using saturation-recovery sequences at 3T [16] and the inter-subject variability (demonstrated by the larger interquartile range in Fig. 6) was greater than for MOLLI as expected due to the lower precision of SASHA-based methods. Although we are unaware of native T1 measurements in the RV myocardium using saturation-based methods at 3T, native SASHA T1 values in the LV at 3T have been reported by Weingärtner et al. (septum = 1523 ± 56 ms, LV = 1500 ± 66 ms) [16] and by Guo et al. (septum = 1567 ± 57 ms, LV = 1560 ± 97 ms) (mean ± standard deviation) [18]. STEAM-SASHA provides a slightly lower mean T1 in the LV (1360 ± 116 ms) and septum (1422 ± 102 ms). While a direct comparison between STEAM-SASHA and a standard SASHA acquisition will be required to determine whether this is a systematic bias in STEAM-SASHA T1 values, a SASHA sequence was not available for our hardware at the time of this study. One contribution to this potential systematic error is the efficiency of the saturation pulse used. Chow et al. [19] developed an optimised 6 pulse design for robust saturation at 3T for SASHA, resulting in higher T1 values than when using a similar 90°–90°–90° pulse design to the one used here, but an effective version of the 6-pulse saturation module was not available for our hardware at the time of this work. It is also possible that blood (which has a longer T1 than myocardium [20]) within the myocardium increases the measured myocardial T1 for more standard SASHA techniques, but not for STEAM-SASHA where the blood signal is suppressed. While not statistically significant (Fig. S6) our findings of lower measured T1 in the RV regions using STEAM-SASHA than MOLLI are consistent with reduced blood signal contamination of the RV signal in STEAM-SASHA data.

In line with other SASHA studies [16, 17], this initial study of STEAM-SASHA suggests the new method is less reproducible and less precise than MOLLI in most myocardial regions. However, our results suggest that in regions such as the anterolateral RV STEAM-SASHA T1 values potentially benefit from the blood and fat signal nulling provided by STEAM-SASHA, resulting in a smaller spread in the Bland–Altman plots when compared to MOLLI, although some improvements in MOLLI reproducibility might be expected with more sequence repeats. The reduced reproducibility and precision of STEAM-SASHA T1 values is likely a consequence of a combination of the reduced dynamic range of saturation recovery-based methods compared to inversion recovery and the multiple sequence repeats, which require additional image registration and regions of interest to be drawn. Small regions of high T1 visible in the examples shown in Fig. 6 are thought to be a consequence of imperfect registration and might be reduced by affine or fully non-rigid registration.

Other studies measuring T1 in the RV myocardium have used standard MOLLI sequences acquired at peak systole [21–23] with the associated effects of blood and fat contamination. Mehta et al. [7] used a navigator gated segmented acquisition with undersampling and compressed sensing reconstruction to increase the spatial resolution of MOLLI T1 mapping data for application in the RV myocardium. Here we chose to address the contaminating blood and fat signals rather than spatial resolution. Pagano et al. [24] used fat–water separation to provide water-only saturation recovery based T1 maps in a single subject at 1.5 T. Heng et al. [6] added motion sensitising gradients to a similar sequence to acquire dark blood, fat suppressed SASHA-like free-breathing T1 data at 1.5T. Results were shown in healthy volunteers and tetralogy of Fallot patients both pre and post-contrast agent administration, although the motion sensitisation required subject specific optimisation. Future work may consider combining STEAM-SASHA with interleaved spiral trajectories, which have been used to improve the spatial resolution of STEAM DT-CMR [25], therefore addressing both the required improvements in isolation of myocardial signal and spatial resolution for RV T1 mapping.

While STEAM provides excellent blood and fat suppression, the low SNR that is inherent to stimulated echoes contributes to a reduction in T1 precision and means that averaging is important to the quality of STEAM-SASHA T1 data, resulting in long acquisitions. This necessity of averaging STEAM data is well known in DT-CMR [26] where, for the resolution used here, 8 averages per slice and diffusion encoding direction would be typical. The use of a saturation pulse reduced the SNR of the TS = 300 ms images further, but the diffusion weighting of the spoiler gradients used in this work (*b* = 100 smm^−2^) is substantially reduced compared to the diffusion weighting typical in DT-CMR (*b *~ 600 s mm^−2^) [27], which similarly reduces the SNR. As a result, for the work presented here the number of sequence repeats was matched to the 8 averages often used in DT-CMR studies, and using fewer sequence repeats was found to result in less precise STEAM-SASHA T1 data. While the current implementation of STEAM-SASHA uses 16 breath holds, which may limit its clinical utility to studies in healthy volunteers and motivated clinical research patients, fewer breath-holds may be enabled using a single EPI navigator and parallel imaging reference dataset for all imaging data or using strategies such as Beltrami [28] or deep learning based denoising [29].

Here STEAM-SASHA was used with a fixed TS = 300 ms saturation delay. The use of longer saturation times would produce higher SNR saturation weighted images and potentially help to address the lower precision of STEAM-SASHA data than the MOLLI data used as a comparison. Using additional saturation delays would also allow more accurate 3 parameter fitting of T1 values to be performed, at least partially accounting for any imperfect saturation. Work by others [30] has suggested that a fixed TS = 591 ms provides slightly more precise native myocardial mapping using SASHA than TS = 290 ms. However, TS = 300 ms allows STEAM-SASHA to acquire the imaging data near end-systole. Future work may improve precision by running the saturation pulse in the prior cardiac cycle, potentially improving T1 precision but requiring a correction in the T1 calculation for the heart rate dependent saturation delay. End-systolic imaging was chosen as the thicker myocardium means that the T1 values have the lowest vulnerability to partial volume effects [22, 31, 32] and is also more robust to variations in RR interval [33, 34]. Furthermore, the acquisition of noise-only images enabled us to demonstrate that the myocardial signal intensity in the TS = 300 ms data is > 4× times above the noise floor in the myocardium and is therefore sufficient for analysis without substantial noise floor effects.

Using the noise-only data we were also able to demonstrate complete suppression of the RV blood pool in the TS = 300 ms and excellent suppression in the anchor images where the myocardial signal intensity was >19 times higher.

STEAM stores the partially prepared magnetisation for 1 cardiac cycle (the mixing time, TM) during which time T1 relaxation occurs and the magnetisation available to create a stimulated echo decays. Variations in heart rate would result in changes in the magnetisation available for imaging. While variations in heart rate between breath holds are expected to result in minimal errors as each breath hold contains both anchor and saturation weighted data images, variations within a breath hold are more likely to cause errors. The effective diffusion weighting of the method also depends on the heart rate as the diffusion encoding time is 1 cardiac cycle. Intra-breath hold heart rate variations would therefore result in errors in measured T1 due to changes in diffusion weighting with heart rate. The effects of heart rate variability induced errors could be reduced in future studies using post-processing based intensity correction for T1-related signal loss and diffusivity.

Here, we have only considered native T1 measurements. Extracellular volume fraction estimations would require the acquisition of additional data, as STEAM-SASHA is intrinsically a dark blood technique. Post-contrast myocardial T1 measurement with STEAM-SASHA would be hampered by a reduction in SNR relative to pre-contrast measurements due to the shorter post-contrast T1 causing additional loss of magnetisation during TM which would not be completely recovered by the additional T1 recovery that would occur between sequence repeats.

Future studies will look to evaluate the performance of STEAM-SASHA in providing measures of native right ventricular myocardial T1 in patient populations, such as repaired tetralogy of Fallot for detection of diffuse fibrosis.

In conclusion, we have introduced the STEAM-SASHA sequence, a saturation recovery prepared STEAM method that provides excellent blood and fat suppression for accurate and reproducible T1 assessment in the right ventricle. The work has illustrated the performance of STEAM-SASHA both in phantoms and in vivo and initial results suggest that this novel method may be less affected by confounding factors in the RV at the expense of some reduction in precision and reproducibility when comparing to MOLLI. STEAM-SASHA therefore, has the potential to provide new insights into pathological RV changes, where time permits.

### Supplementary Information

Below is the link to the electronic supplementary material.

Supplementary file1 (PDF 6494 kb)

## Data Availability

The data of all the experiments is available from the corresponding author on reasonable request subject to ethical approval and data sharing agreements.
