# Supplementary material for: STEAM-SASHA: a novel approach for blood- and fat-suppressed native T1 measurement in the right ventricular myocardium
Source: MAGMA. 2024 Jan 12;37(2):295–305. doi: 10.1007/s10334-023-01141-8 (PMC10995026; doi:10.1007/s10334-023-01141-8)
Supplement: Supplementary file 1 — Supplementary file1 (PDF 6494 kb) [file 10334_2023_1141_MOESM1_ESM.pdf]

Supplementary material

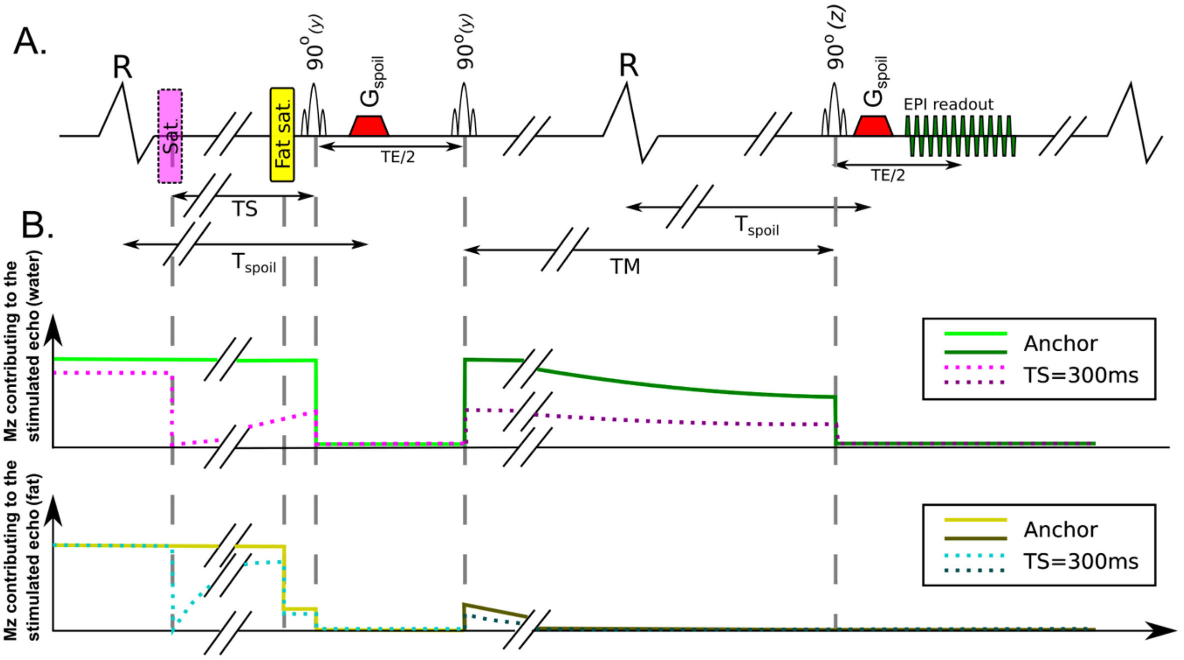

Supplementary figure 1: The evolution of the longitudinal magnetisation available to generate a stimulated echo (Mz) during the STEAM-SASHA sequence for both the anchor and TS=300ms acquisitions, both for water and fat protons. For abbreviations and further details see figure 1 in the main manuscript.

The magnetisation is shown in a different colour after the second RF pulse to denote the fact that we do not show the component of the magnetisation that is not stored to generate the stimulated echo, but recovers to generate a free induction decay signal after the third RF pulse that is spoilt by G_{spoil} before the EPI readout. The majority of the fat signal is saturated by the spectrally selective fat saturation pulse (fat sat) and therefore does not contribute to the stimulated echo despite almost full recovery before the third RF pulse due to the second spoiler gradient G_{spoil} that eliminates the free induction decay signal. Any residual fat magnetisation that is not saturated by the fat saturation pulse and would contribute to the stimulated echo recovers towards equilibrium with T1 during the mixing time. The rapid recovery to equilibrium of this residual fat signal due to its short T1 results in no observable residual fat in the final stimulated echo images.

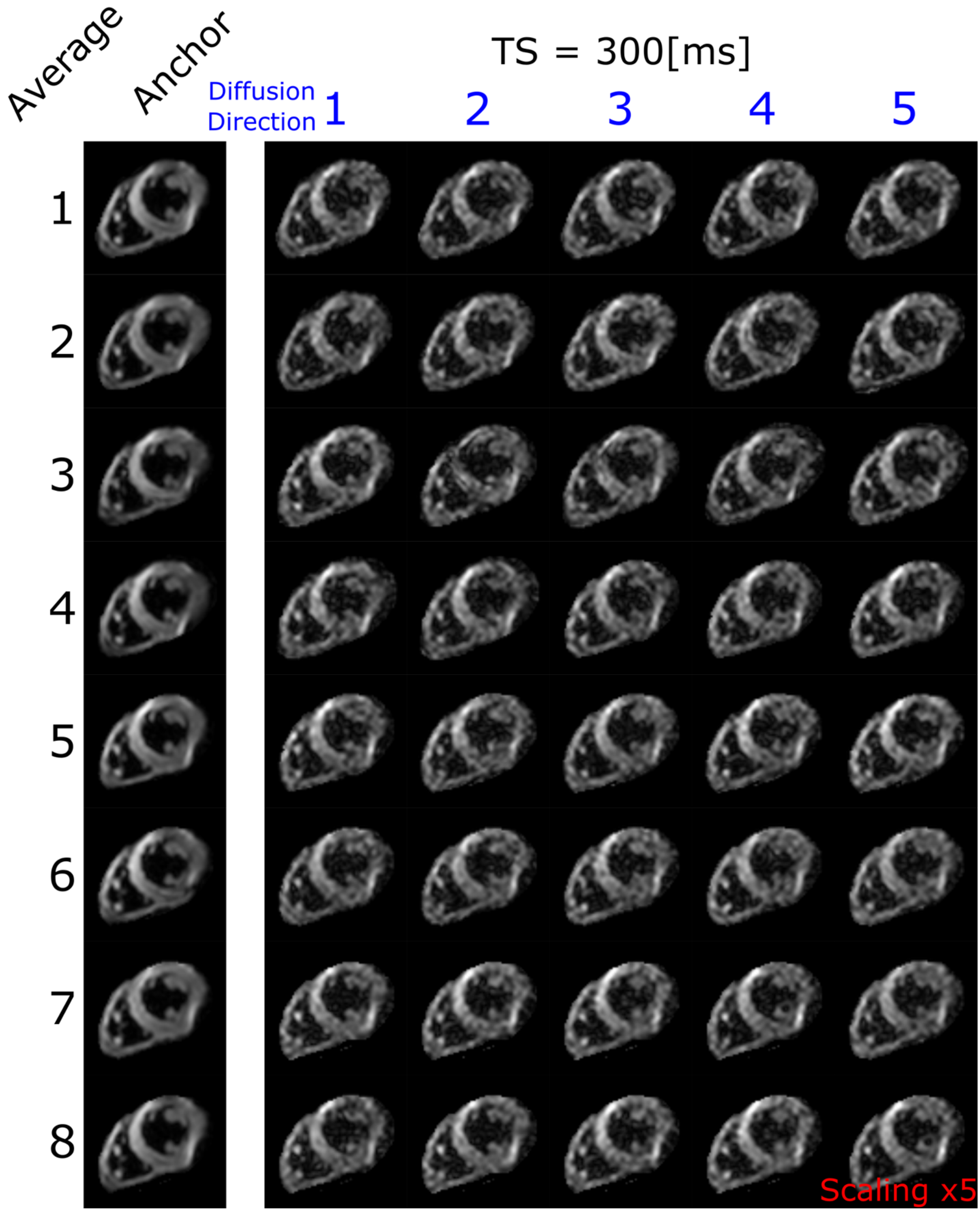

Supplementary figure 2: Example STEAM-SASHA images shown before signal averaging in a subject where data quality was considered poor. Images for TS=300ms have been scaled x5 in brightness. Signal outside the heart has been cropped. Each average corresponds to a separate pair of breath holds. The effective diffusion encoding magnitude was low (b=100smm^{-2}), but the encoding direction was varied during each breath hold to avoid regional bias in calculated T1 due to SNR differences.

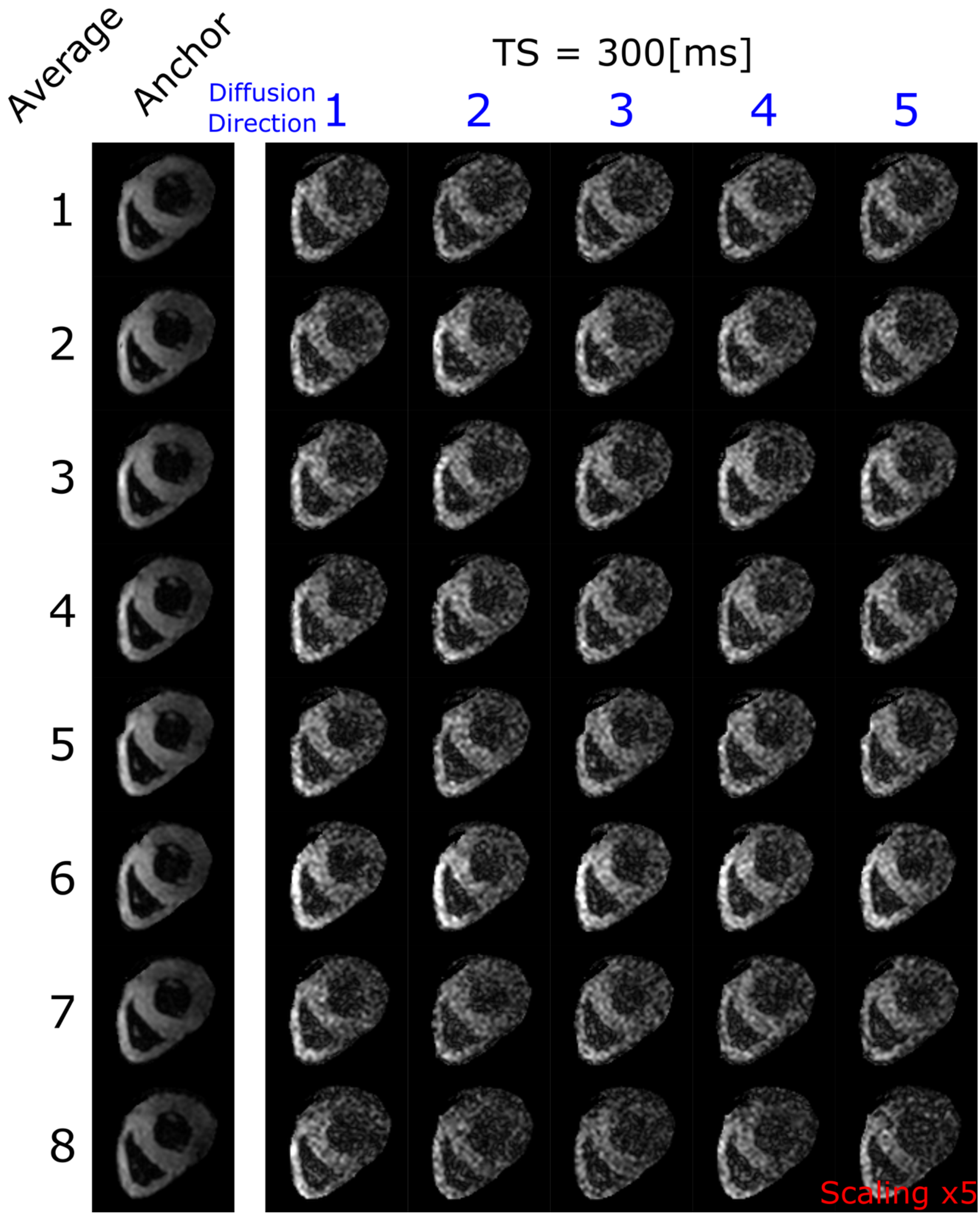

Supplementary figure 3: Example STEAM-SASHA images shown before signal averaging in a subject where data quality was considered typical. For details see the supplementary figure 2 caption.

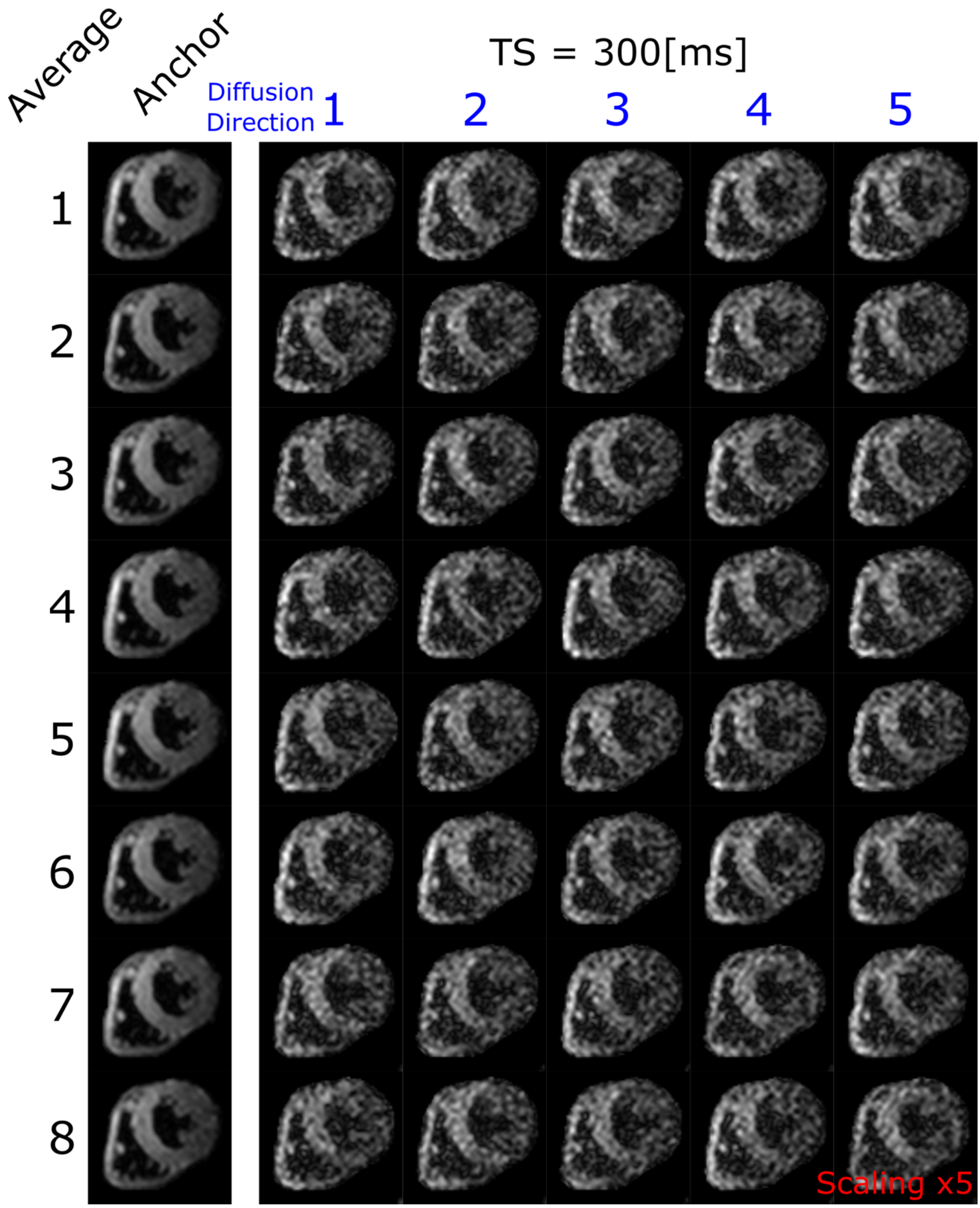

Supplementary figure 4: Example STEAM-SASHA images shown before signal averaging in a subject where data quality was considered high. For details see the supplementary figure 2 caption.

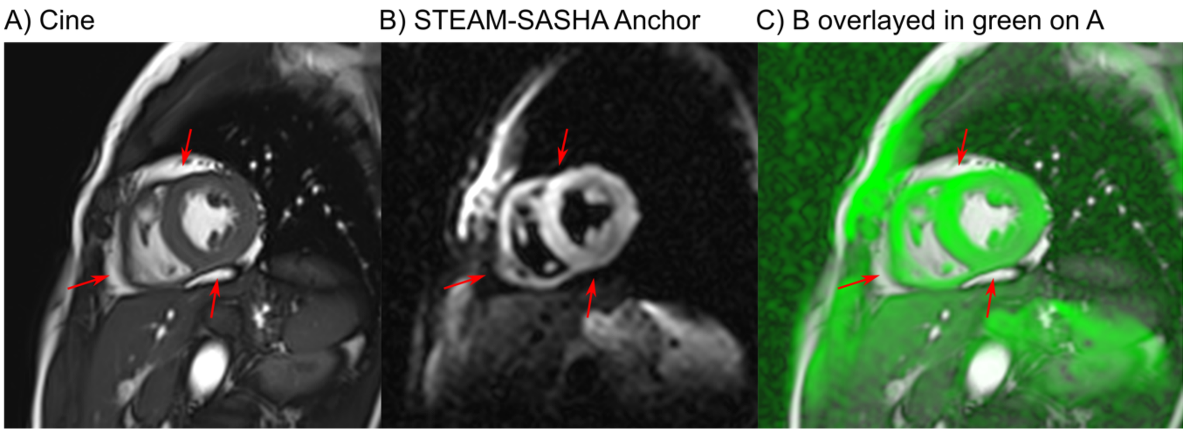

Supplementary figure 5: STEAM-SASHA provides excellent suppression of fat signal. This example shows data from the subject with the most epicardial fat of the 10 imaged in this study. The cine data (A) clearly shows the high signal in the epicardial fat surrounding the myocardium (red arrows), which is non-existent in the equivalent STEAM-SASHA anchor image (B). An overlay of the STEAM-SASHA data colourised green on top of the cine data from A, further highlights the absence of fat in the STEAM-SASHA data (C).

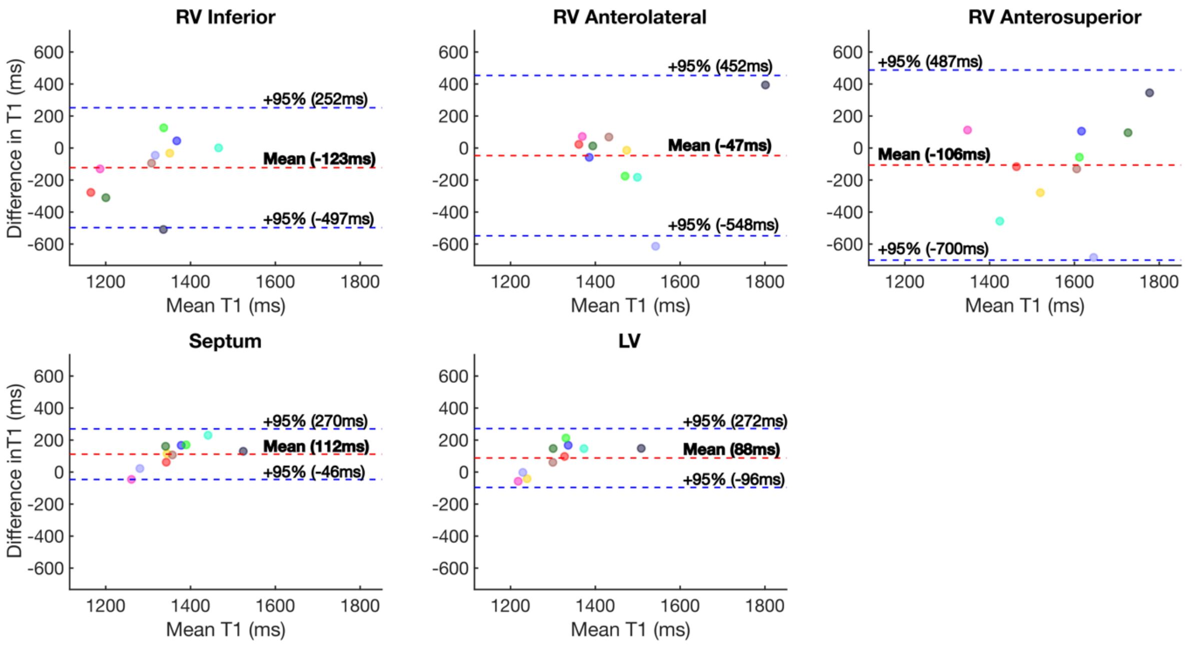

Supplementary figure 6: Bland-Altman plots for the STEAM-SASHA vs MOLLI data. Each of the 10 subjects is plotted in a different, consistent colour. The difference is calculated as the STEAM-SASHA T1 minus the MOLLI T1. The dotted blue line marks the 95% confidence interval of the difference, while the red dotted line is the mean difference between repeats (bias). In the septum and LV, a significant positive correlation is observed in the difference in T1 and mean T1 (R=0.69, p=0.03 and R=0.70, p=0.02 respectively). Correlations in the other regions were not significant (p>0.05).

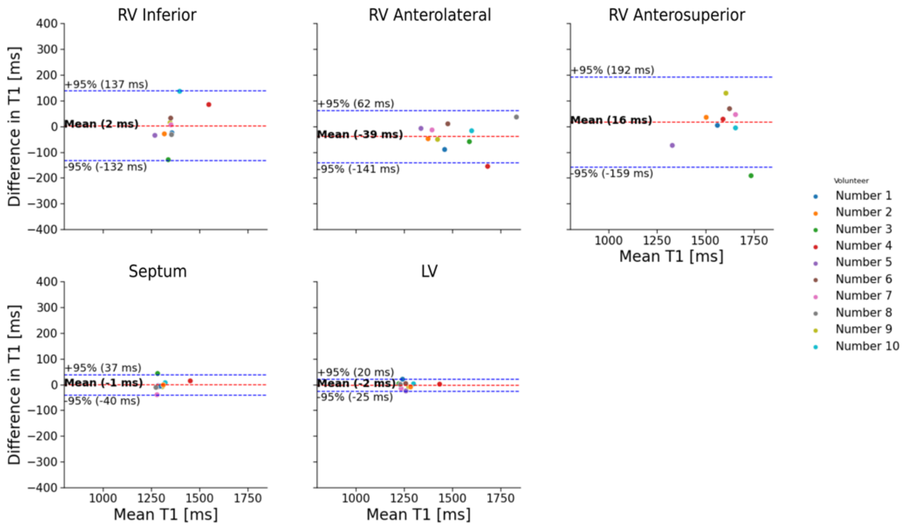

Supplementary figure 7: Inter-study Bland-Altman plot for the MOLLI sequence for different myocardial regions. For each of the 10 volunteers (each plotted in a different colour), two repeated scans were acquired with subject repositioning performed between acquisitions. The plot shows the difference in T1 between acquisitions vs. the mean T1 over both repeats. The dotted blue line marks the 95% confidence interval of the difference, while the red dotted line is the mean difference between repeats (bias).

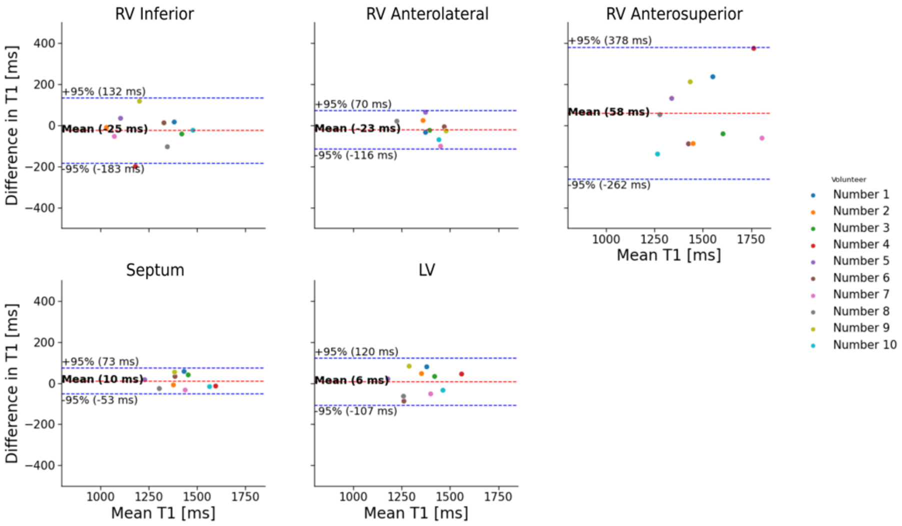

Supplementary figure 8: Inter-study Bland-Altman plot for the STEAM-SASHA sequence for different myocardial regions. For detailed description see supplementary figure 7.

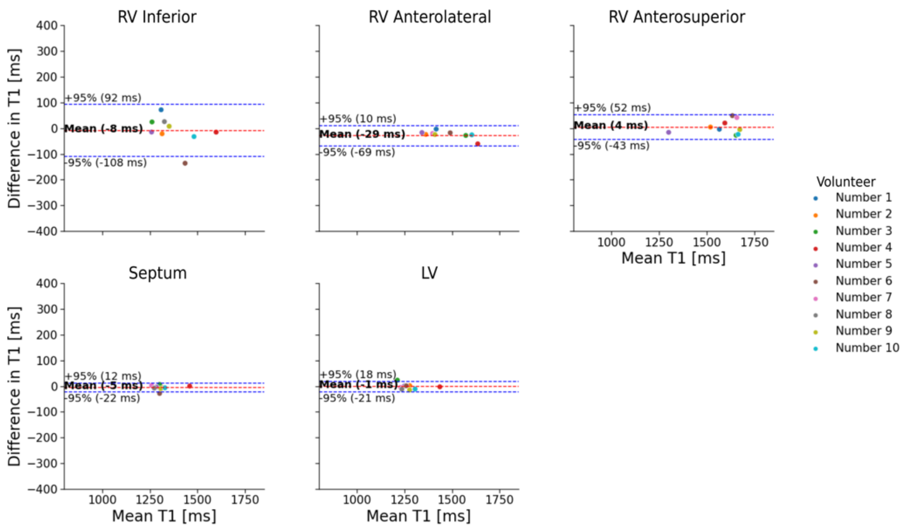

Supplementary figure 9: Intra observer Bland-Altman plot for the MOLLI sequence for different myocardial regions. For each of the 10 volunteers (each plotted in a different colour), the same image reader performed the analysis spaced 2 weeks apart. The plot shows the difference in T1 between analyses vs. the mean T1 over both repeats. The dotted blue line marks the 95% confidence interval of the difference, while the red dotted line is the mean difference between repeats.

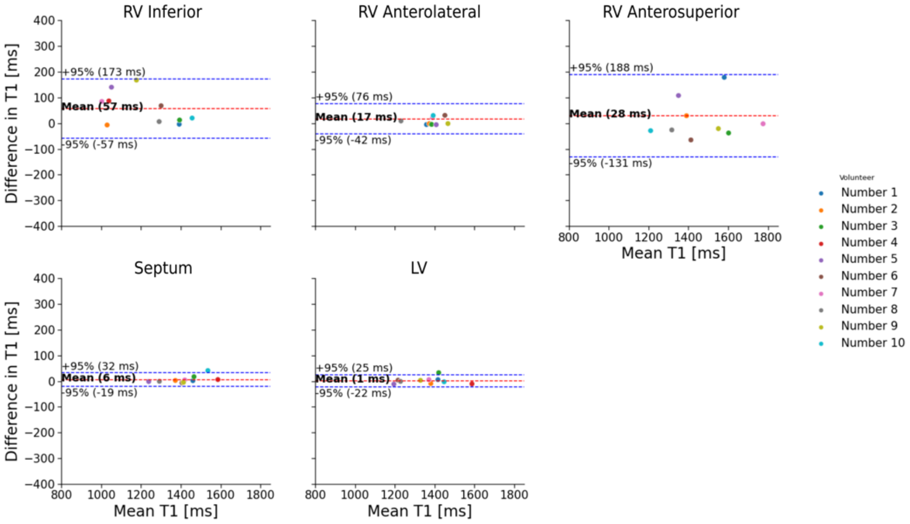

Supplementary figure 10: Intra observer Bland-Altman plot for the STEAM-SASHA sequence for different myocardial regions. For detailed description see supplementary figure 9.

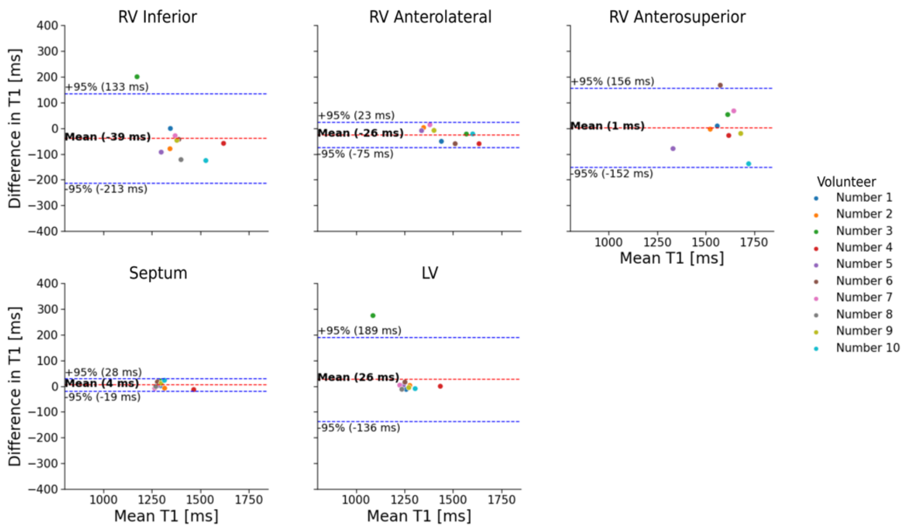

Supplementary figure 11: Inter observer Bland-Altman plot for the MOLLI sequence for different myocardial regions. For each of the 10 volunteers (each plotted in a different colour), two different image readers performed the analysis. The plot shows the difference in T1 between analyses vs. the mean T1 over both repeats. The dotted blue line marks the 95% confidence interval of the difference, while the red dotted line is the mean difference between repeats.

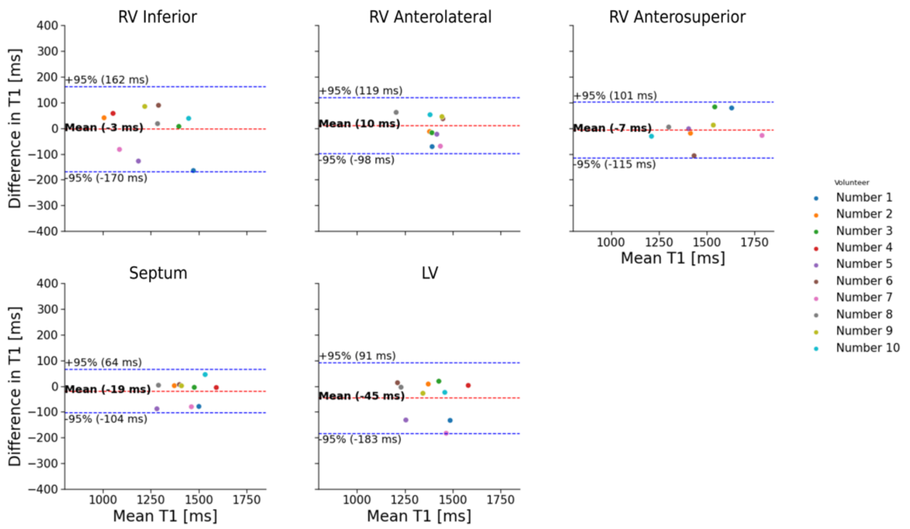

Supplementary figure 12: Inter observer Bland-Altman plot for the STEAM-SASHA sequence for different myocardial regions. For detailed description see supplementary figure 11.

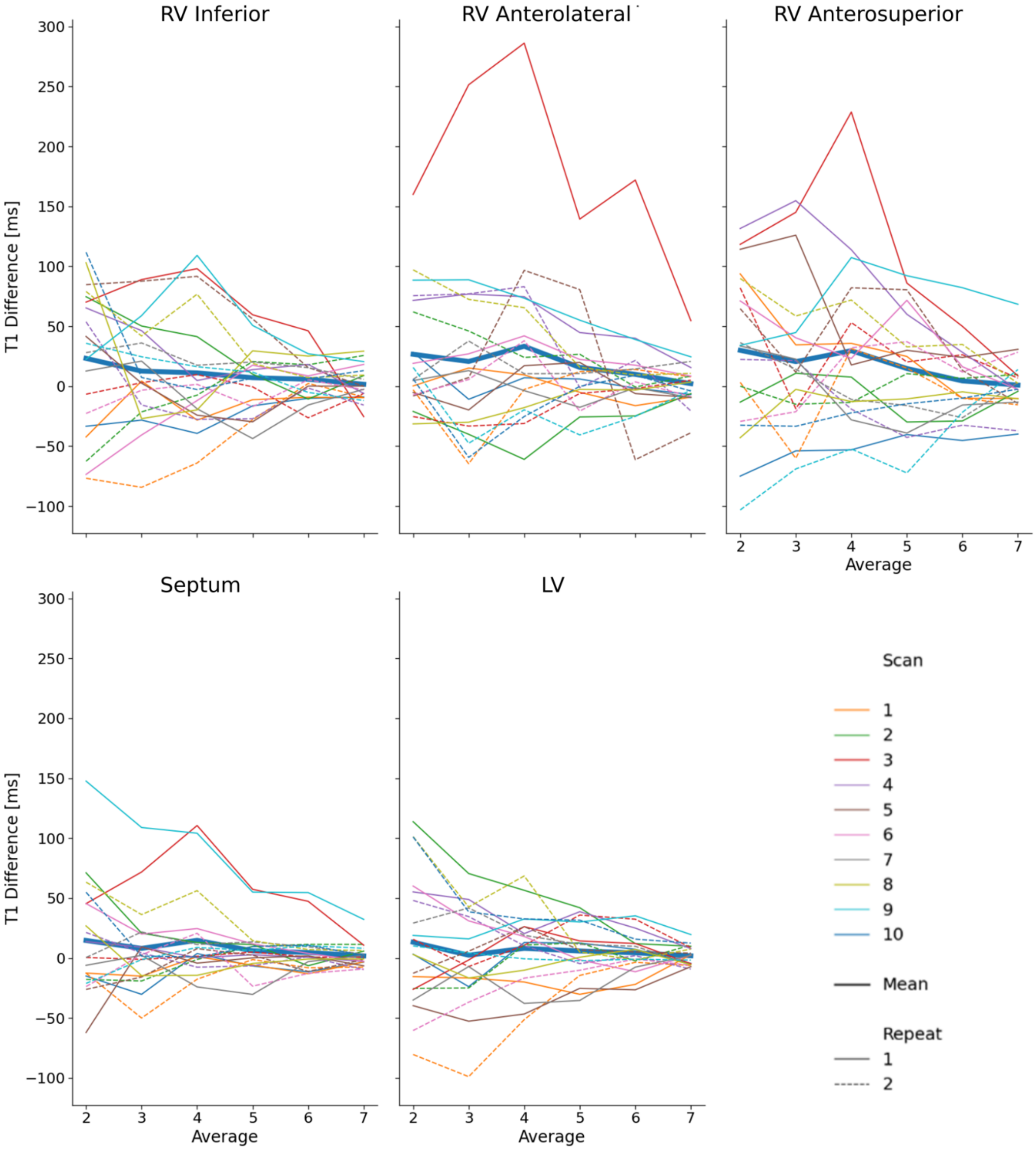

Supplementary figure 13: The mean difference in T1 with an increasing number of averages for the 5 myocardial regions using 8 averages as the reference. Since each subject was scanned twice the second repeat is shown as a dotted line. The mean of all subjects and repeats is shown as a thicker blue line.
